# Supplementary material for: Protective Role of p66Shc Deletion in Physiological Renal Aging: Effects on G Protein-Coupled Receptor 124 Expression and Associated Cellular Senescence
Source: Int J Mol Sci. 2025 Nov 17;26(22):11096. doi: 10.3390/ijms262211096 (PMC12652738; doi:10.3390/ijms262211096)
Supplement: Supplementary file 1 [file ijms-26-11096-s001.zip › ijms-3990895-supplementary.pdf]

**Table S1. Antibodies used in Western blot, IHC, and IF studies.**

| Target | Antibody | Catalog Nr. | Supplier |
|---|---|---|---|
| **Primary** | | | |
| COL-IV (IHC) | Rabbit polyclonal | ab6586 | Abcam, Cambridge, UK |
| WT1 (IF) | Goat polyclonal | sc-15421 | Santa Cruz Biotechnology, Dallas, TX, USA |
| NOX4 (IHC) | Rabbit monoclonal | ab133303 | Abcam, Cambridge, UK |
| Nitrotyrosine (IHC) | Rabbit Polyclonal | ab42789 | Abcam, Cambridge, UK |
| GPR124 (IHC-WB) | Rabbit polyclonal | ab198817 | Abcam, Cambridge, UK |
| $p16^{INK4a}$ (IHC-WB) | Rabbit polyclonal | ab211542 | Abcam, Cambridge, UK |
| β-actin (WB) | Mouse monoclonal | MA1-140 | Invitrogen, Thermo Fisher Scientific, Waltham, MA, USA |
| **Secondary** | | | |
| COL-IV, NOX4, GPR124 (IHC), $p16^{INK4a}$ (IHC) | Biotinylated goat anti-rabbit IgG | E0432 | Agilent Dako, Santa Clara, CA, USA |
| GPR124 (WB), $p16^{INK4a}$ (WB) | HRP-conjugated goat anti-rabbit | A16096 | Invitrogen, Thermo Fisher Scientific, Waltham, MA, USA |
| β-actin (WB) | HRP-conjugated goat anti-mouse | ab205719 | Abcam, Cambridge, UK |

COL-IV = Collagen IV; IHC = immunohistochemistry; WT1 = Wilms tumor 1; IF = immunofluorescence; NOX4 = NADPH ossidasi 4; GPR124 = G-protein coupled receptor 124; WB = Western blot; $p16^{INK4a}$ = protein16 inhibitor of cyclin-dependent kinase 4.

**Table S2. TaqMan Gene Expression assays.**

| Target | Assay ID |
|---|---|
| *Col4a1* | Mm01210125_m1 (# 4331182) |
| *Tgfb1* | Mm01178820_m1 (# 4331182) |
| *CDKN2A* | Hs00923894_m1 (# 4331182) |
| *CDKN1A* | Hs00355782_m1 (# 4331182) |
| *CDKN2D* | Hs00176481_m1 (# 4331182) |

*Col4a1* = collagen type IV alpha 1; *Tgfb1* = transforming Growth Factor Beta 1; *CDKN2A* = cyclin-dependent kinase inhibitor 2A; *CDKN1A* = cyclin-dependent kinase inhibitor 1A*;* *CDKN2D* = cyclin dependent kinase inhibitor 2D.

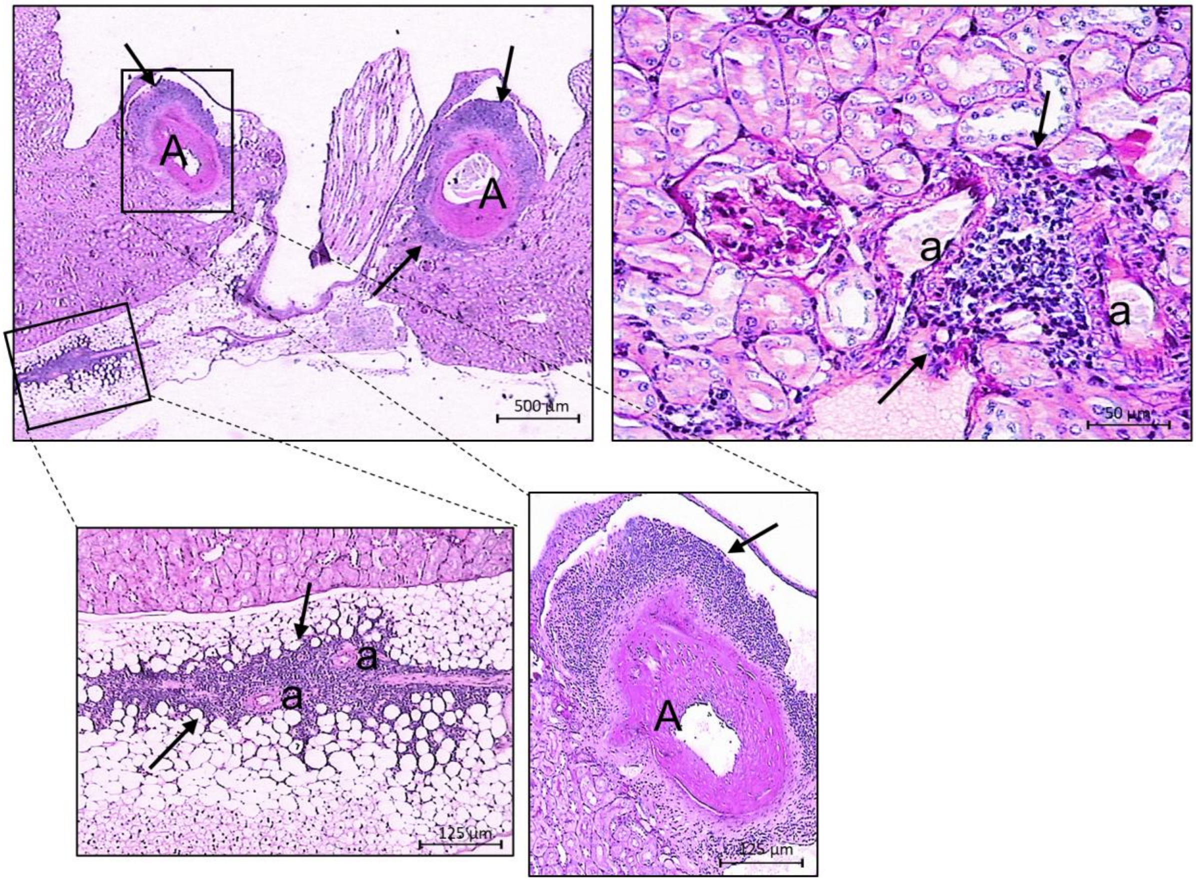


**Supplementary Figure S1**. **Inflammatory cell infiltration surrounding renal vessels in the cortex and hilum**. PAS-stained renal sections showing perivascular inflammatory infiltrates surrounding renal arteries and arterioles in the cortex and renal hilum, consistent with active infection or immune/inflammatory response. Arrows indicate areas of inflammatory infiltrate; A/a = Artery/arteriole.
